# A Usability Pilot Study of a Sensor-Guided Interactive System for Dexterity Training in Parkinson’s Disease

**DOI:** 10.3390/s25041051

**Published:** 2025-02-10

**Authors:** Nic Krummenacher, Stephan M. Gerber, Manuela Pastore-Wapp, Michael Single, Stephan Bohlhalter, Tobias Nef, Tim Vanbellingen

**Affiliations:** 1 Gerontechnology and Rehabilitation Group, ARTORG Center for Biomedical Engineering Research, University of Bern, 3012 Bern, Switzerland; nic.krummenacher@unibe.ch (N.K.);; 2 Neurocenter, Luzerner Kantonsspital, University Teaching and Research Hospital, University of Lucerne, 6000 Lucerne, Switzerland; 3 Department of Neurology, University Hospital Zurich, 8091 Zurich, Switzerland; 4 Department of Neurology, Inselspital, 3010 Bern, Switzerland; 5 VAMED Management & Services Schweiz AG, Research and Innovation, 8001 Zürich, Switzerland

**Keywords:** dexterity, Parkinson’s disease, usability, fine motor skills, gyrosope, accelerometer, force resistive sensors, exergaming, interactive

## Abstract

This pilot study aimed to evaluate the usability of a new, feedback-based dexterity training system in people with Parkinson’s disease (PwPD) and healthy adults. Seven PwPD and seven healthy adults participated in the study. The System Usability Scale (SUS) and the Post-Study System Usability Questionnaire Version 3 (PSSUQ) were used to assess usability. Additionally, the feedback shown as a counter, detected through newly developed algorithms, was evaluated by comparing the device-detected repetitions during six exercises to those counted by a supervisor. High median SUS scores of 92.5 were obtained in both PwPD (IQR = 81.25–98.75) and healthy adults (IQR = 87.5–93.75, maximum score 100, minimum score 0). Similarly, high PSSUQ median scores were achieved after the session (1.14, IQR = 1.00–1.33, PD; 1.08, IQR = 1.00–1.58, healthy adults, maximum score 1, minimum score 7). PwPD completed 648 repetitions, with 551 (85%) correctly recognized by the system. For healthy adults, 883 out of 913 (97%) repetitions were classified as right. The present study showed high usability and high perceived user satisfaction for the new training system in all study participants. The system effectively detects exercise repetition rates but requires further refinement to enhance accuracy for specific pinch grip exercises.

## 1. Introduction

Parkinson’s disease (PD) is one of the most prevalent neurodegenerative disorders, affecting 10 million people worldwide [1,2]. The disorder features both motor (e.g., bradykinesia, rigor, and tremor) and non-motor symptoms (e.g., cognitive impairment, hyposmia, and depression) that can affect functional abilities and quality of life [3,4]. Rehabilitative interventions, including physical, occupational, and speech therapy, are essential in PD’s care, significantly enhancing health outcomes [5]. A growing body of evidence shows that these interventions improve both motor and non-motor functions, support activities of daily living (ADL), and boost quality of life for people with Parkinson’s disease (PwPD) [1,6,7]. In the early stages of the disease, PwPD typically receive rehabilitative interventions in an outpatient setting. When care in this setting becomes too complex, PwPD are often referred to a specialized Parkinson’s clinic or center, where they can access multidisciplinary therapy [5]. Upon discharge, many continue with outpatient therapy supplemented by home-based exercises [8,9].

In recent years, the use of telehealth for therapy, including video calls and telemonitoring, has expanded considerably, a trend further accelerated by the COVID-19 pandemic [10]. Given the progressive nature of PD and the nearly normal life expectancy [11], exercise programs need to be sustainable. Home-based, prescribed exercise offers a practical approach that can be maintained long-term with minimal resources [8]. It has proven effective in reducing overall motor symptoms [12] and enhancing balance [8] and dexterity [9], provided that the excercise is consistently followed. However, adhering to the training protocol can be challenging due to the absence of close supervision and immediate feedback for performance adjustments [13]. Additionally, the training methods may become monotonous, further hindering long-term commitment. Technological advances in sensor-based devices and game-based applications, often referred to as exergames, offer promising solutions for enhancing motivation and adherence in home-based exercise rehabilitation programs [14,15]. Specifically, exergames combine physical exercise with interactive video games [16], requiring patients to move specific body parts, such as the hands or fingers, to generate corresponding responses within the game. One approach for dexterity training in PD using exergames involves a motion-tracking device (Leap Motion) that captures hand and finger movements, allowing real-time in-game responses to measure exercise performance [17,18]. However, this device lacks tactile feedback, which could further improve the training experience. Adding tactile feedback while manipulating the device using the fingers is expected to provide more precise and functional movement training relevant to ADL [19].

A recently developed device, known as the Smart Sensor Egg (SSE) [20], is a pocket-sized, sensor-based tool designed to enhance dexterity (coordinated finger movements) and hand function (grip and grasp), which are often impaired in PwPD [21,22] and the healthy elderly [23]. Good hand function and dexterous skills are critical for performing activities of daily living (ADL), such as cooking, buttoning clothes, or writing [22,24,25]. In an initial study, the first prototype of the SEE, equipped with a gyroscope, an accelerometer, and a force sensing resistor (FSR), was evaluated [20]. Participants, supervised by a clinician, successfully used the device, which was connected to a laptop running a single game. The device was well-received, and the gamified environment proved motivating for training hand functions. However, to enable the use of this innovative device in a home-based setting, further development is required, both in the technical design of the device and the accompanying game-based software (v1).

The aim of this study is to evaluate a new prototype of the Smart Sensor Egg, which incorporates more advanced technical features and a newly developed, comprehensive software (v2) application (app) installed on a smartphone. Unlike the previous version, this updated setup, combined with the new training software (v2), enables unsupervised dexterity training at home by providing feedback on the number of repetitions completed during exercises. To achieve this, new algorithms have been developed to analyze how the Sensor Egg is manipulated through hand and finger movements, ensuring the precise recognition of motion patterns. This recognition is essential for making unsupervised training more interactive and engaging by continuously adapting feedback based on the user’s movements. Before launching a larger prospective training program at home, we evaluate whether the developed algorithms can detect exercise repetitions, provide appropriate feedback as a repetition count, and gauge user acceptance of the new software (v2). We hypothesize that the newly developed algorithms will detect repetitions and that the app will achieve high usability and satisfaction thanks to its clear and straightforward instructions.

## 2. Materials and Methods

### 2.1. Recruitment and Participants

A convenience sample of PwPD and healthy adults was recruited at the Cantonal Hospital Lucerne. PwPD were eligible for inclusion in the study if they had a diagnosis of PD as defined by the UK Parkinson’s Disease Society Brain Bank Criteria [26], were aged between 45 and 85 years, reported subjective difficulties with dexterity, had a Montreal Cognitive Assessment (MoCA) [27] score greater than 21, and did not have excessive or uncontrollable upper limb tremor. In the case of healthy people, age and gender-matched participants were recruited. Written informed consent was obtained, and the Declaration of Helsinki was adhered to. Ethical approval was granted by the Ethics Committee of the Canton of Lucerne, Switzerland (BASEC ID 2022-02300).

### 2.2. The Smart Sensor Egg System

**Figure 1:**
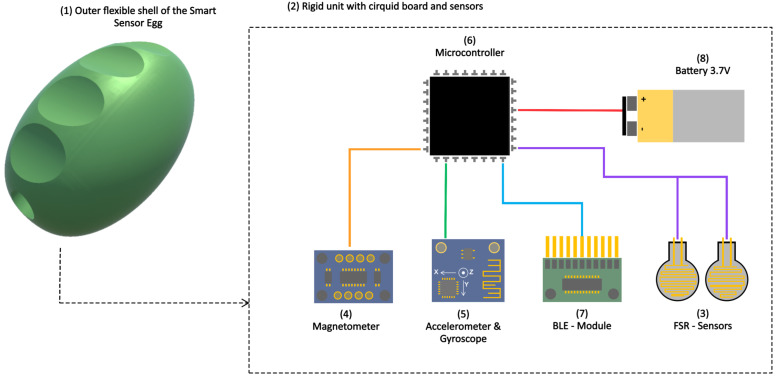
The sensing part (2) of the Smart Sensor Egg (SSE) is encapsulated by a flexible outer shell (1). It consists of two force sensing resistors (FSRs) (3), a magnetometer (4), and a tri-axis gyroscope and a tri-axis accelerometer (5). A microcontroller (6) with a Bluetooth low energy (BLE) module (7) is used to process and stream data to the smartphone app. A 3.7V lithium battery (8) is used to power the system.

**Figure 2:**
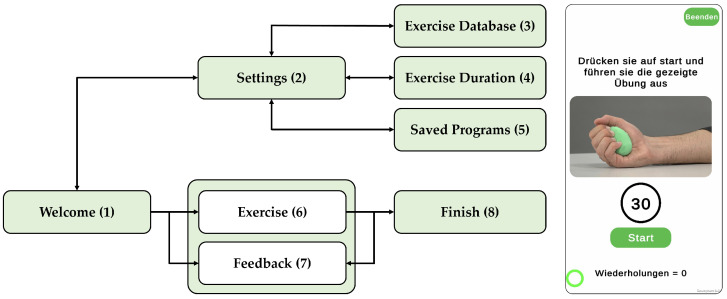
The smartphone app is structured to follow an event-driven principle of interacting with the different states presented on eight screens. After opening the app, the welcome screen (1) automatically connects to the SSE. The settings (2) allow the user to create a training program from a database of six different exercises (3), select the duration of each exercise (4), and save and recall different training programs (5). After starting the training program, the exercise screen (6) displays a video instruction of the current exercise, a countdown that starts when the start button is pressed, and a counter showing the number of repetitions recognized by the Algorithms. If the exercise is performed incorrectly or is not recognized, the counter will not increase. At the end of the countdown, the application switches to the feedback screen (7), which shows the number of repetitions performed and compares it with the performance of the previous training session. The application will continue with the next exercise until the training program is completed in the finish screen (8).

The earlier version of the SSE used by Saric et al. [20] was enhanced for hand dexterity and coordination of individual fingers. This improved version was utilized in the present study. The new SSE system consists of a hardware device (Figure 1) and a smartphone app (Figure 2) designed for unsupervised dexterity training. The hardware features a flexible, hand-sized, egg-shaped outer shell that deforms under pressure and encloses a rigid inner unit, housing a circuit board. The SSE system’s smartphone app facilitates feedback-driven home training by guiding users through structured exercises with adjustable durations and guided video instructions. In addition, the app provides real-time feedback based on SSE sensor data, displayed as a repetition counter. The SSE system’s smartphone app was developed using Unity 3D (Unity Technologies, San Francisco, CA, USA), Editor Version 2021.3.0f1.

### 2.3. Experimental Procedure

Participants were given an SSE and a smartphone. Prior to the start of the intervention, they were instructed by the supervisor not to ask any questions during the session to simulate a home-like environment where they would perform the tasks independently. Participants completed a series of tasks, including a training session comprising six exercises conducted with the SSE system. The tasks were guided by written instructions (Supplementary Files). After completing the tasks, they were asked to complete usability and satisfaction questionnaires. The entire procedure took approximately 35 min per participant.

### 2.4. Evaluation

To assess the potential usability of the training system in a home-based environment, the System Usability Scale (SUS) [28] and the Post-Study System Usability Questionnaire Version 3 (PSSUQ) [29] were used.

The SUS is a widely used and well-validated ten-item, 5-point scale ranging from 1 (strongly disagree) to 5 (strongly agree). The SUS scores can range from 0 to 100, with higher scores indicating higher usability. Above a score of 70, the system is considered usable, and above 90, it is considered to have excellent usability [30]. These qualifier words will be used to describe the numeric SUS results. The PSSUQ evaluates users’ perceived satisfaction with computer systems or applications. It comprises a 16-item, 7-point scale ranging from 1 (strongly agree) to 7 (strongly disagree), with an N/A option. The PSSUQ is divided into 3 sub-areas: System Quality (SysQual), Information Quality (InfoQual), and Interface Quality (IntQual). The resulting PSSUQ scores can range from 1 to 7, with a lower score indicating higher satisfaction [29].

Six exercises were selected for the training session to evaluate the algorithms that analyze the sensor data and provide feedback in consultation with occupational therapists experienced in hand rehabilitation from the Lucerne Cantonal Hospital. All exercises typically address hand and finger motor skills (Table 1). The accuracy of the recognition of individual repetitions was examined by counting the individual repetitions manually by the supervisor.

### 2.5. Statistical Analysis

Statistical analysis was performed using R [31] (R Foundation for Statistical Computing, Vienna, Austria), Version 4.4.1, with a significance level of *p* < 0.05. The validity of the normal distribution of the data was tested using the Shapiro–Wilk test. Due to the small number of test participants, no parametric tests were performed. Mann–Whitney (age) and chi-squared tests (sex) were used to test for equivalence between groups with respect to demographic characteristics. Descriptive statistics were used to calculate usability and satisfaction scores. Box plots with scatter were used to visualize PSSUQ scores. Spearman correlation was used to examine the relationships between demographic characteristics (MDS-UPDRS-III, Age), which may influence the overall usability. The number of repetitions for each exercise was calculated across all participants to assess the algorithm’s ability to count the repetitions performed correctly. Exercises performed incorrectly were excluded from the analysis.

## 3. Results

### 3.1. Demographic

A total of seven PwPD and seven healthy participants were recruited. There was no significant difference in age or sex proportions between the two groups. Additional details regarding clinical measures are provided in Table 2.

### 3.2. Usability and Satisfaction

**Figure 3:**
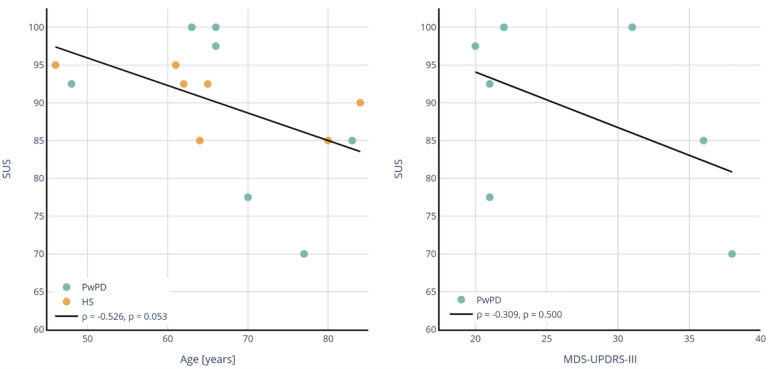
Correlation between age and System Usability Scale (SUS) score and MDS-UPDRS-III and SUS for healthy subjects (HS) and people with Parkinson’s disease (PwPD).

The median usability score was 92.5 for both groups, with an IQR of 81.25–98.75 for PwPD and 87.5–93.75 for the healthy participants, indicating excellent usability. A moderate negative Spearman correlation was observed between age and SUS scores (r = −0.526), with a *p*-value (*p* = 0.053) approaching significance (Figure 3). In contrast, a weak negative correlation was found between UPDRS-III scores and SUS scores (r = −0.309), with no statistical significance (*p* = 0.500).

**Figure 4:**
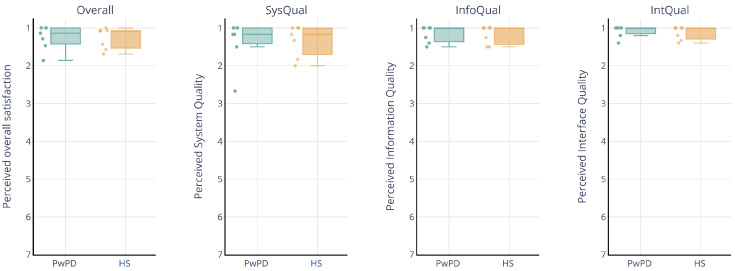
Post-Study System Usability Questionnaire (PSSUQ) overall satisfaction and sub-area scores for seven PwPD and seven healthy subjects (HS).

The total PSSUQ score was 1.14 (IQR = 1.00–1.33) for PwPD and 1.08 (IQR = 1.00–1.58) for healthy participants, indicating high perceived overall satisfaction [29] (Figure 4). Specifically, both groups rated system quality with a median of 1.17 and an IQR of 1–1.33 for PwPD and 1–1.58 for healthy participants. For information quality, both PwPD and healthy participants had a median score of 1, with an IQR of 1–1.32 for PwPD and 1–1.38 for healthy participants. Similarly, for interface quality, PwPD rated a maximum median score of 1 with an IQR of 1–1.27, and healthy participants rated interface quality with a median of 1 and an IQR of 1–1.10.

### 3.3. Algorithm Detection

In exercises one, two, and four, the algorithm detected repetitions less accurately because the SSE was held incorrectly or the force was insufficient to meet the established threshold for correct counting. In exercises five and six, three participants dropped the SSE (Table 3, red). The supervisor counted 1561 repetitions across all six exercises, and all included participants. Of these, 648 were performed by PwPD and 913 by healthy participants. Compared with the exercises recognized by the algorithm, 551 of the 648 (85%) repetitions were correctly recognized in the PwPD and 883 of the 913 (97%) in the healthy participants. A detailed overview of the repetitions counted by the supervisor and repetitions detected by the algorithm of each participant and exercise is provided in Table A1.

## 4. Discussion

This usability study introduced an innovative system that integrates sensor technology with a new application for unsupervised dexterity training. Consistent with our hypothesis, the SSE system demonstrated high usability and satisfaction among both healthy participants and PwPD. Additionally, the system’s algorithms effectively detected repetitions and provided feedback through accurate counts. The high usability aligns with findings from a previous study that evaluated the initial SSE prototype [20] and is consistent with results from other innovative dexterity training systems for PwPD [32,33]. The new app was well received by all users, including both PwPD and healthy subjects, as evidenced by the high satisfaction rates. The findings suggest that while age may have an influence on perceived usability, motor impairment, as measured by MDS-UPDRS-III, does not seem to play a significant role. However, further investigation with larger sample sizes will be needed to validate these observations.

Several factors likely contributed to the high usability and satisfaction ratings of the new SSE and app. First, feedback from users of the initial SSE prototype was incorporated into the updated system, resulting in improvements to the hardware. Second, experienced occupational therapists specializing in hand rehabilitation advised the development team on which core exercises should be incorporated into the app. Third, unlike the previous study, the new app was installed directly on a smartphone, streamlining the setup process and reducing time requirements. Lastly, all users were able to follow a manual independently and were well guided by instructional video’s demonstrating the exercises to be performed with the SSE. The clear instructions may also explain the high scores in the subcategories of the PSSUQ, being information and interface quality. These positive user experiences highlight the potential of the SSE system for effective, unsupervised dexterity training at home.

The newly developed algorithm demonstrated high to nearly perfect repetition detection rates, achieving 85% accuracy in PwPD and 97% in healthy subjects. However, detection rates were lower for PwPD specifically during Exercises 1, 2, and 3. These exercises involved a pinch grip and required fine sensorimotor control, which may have been impaired, as is commonly observed in PD [34]. This finding aligns with recent research indicating that PwPD often struggle with generating submaximal rapid pinch forces [35]. Furthermore, the lower detection rate may also reflect an inherent impairment of proper scaling force in motor control, which relies on intact sensory feedback mechanisms [36]. Moreover, some degree of fatigue may have affected performance and reduced detection accuracy, particularly toward the end of an exercise.

**Figure A1:**
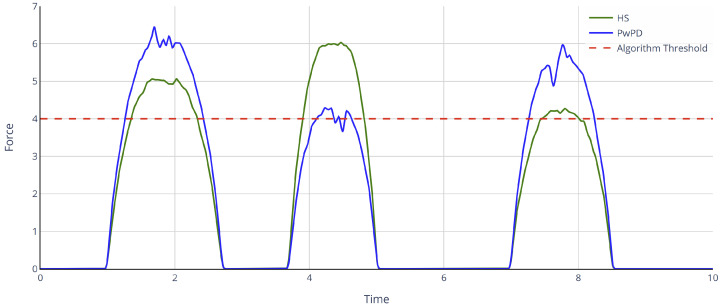
Example signal for exercises one, two, or three. The signal is not derived from a study subject but was created to visualize potential signal patterns. The green line represents how a signal might appear for a healthy participant, while the blue line illustrates a possible signal for an individual with Parkinson’s disease.

Another potential reason could be the interaction between the exercise design and the algorithm’s thresholding approach. Since these exercises required compressing and releasing the SSE with the index finger, many PwPD hovered around the algorithm’s threshold, causing multiple repetitions to be erroneously recorded simultaneously (Figure A1). To address this algorithmic limitation, a potential solution could involve modifying the algorithm to register a new repetition only when the sensors detect a complete release of force after crossing the threshold. This adjustment would enable the more precise counting of the exercise. Furthermore, in several instances incorrect exercise performance may have prevented the algorithm from registering any repetitions. Enhancing the clarity of instructions, particularly regarding proper starting positions and execution techniques, could help reduce these errors. These findings highlight the importance of both refining the algorithm and improving further instructional design to enhance the SSE system’s accuracy and effectiveness in future iterations. For exercises requiring more dexterous or coordinated movements (Exercises 4 to 6), detection rates were consistently high and comparable between PwPD and healthy subjects, indicating that the algorithm is already functioning effectively in these cases.

A limitation of this study was the small sample size, consistent with prior research on novel sensor-based rehabilitation interventions [17,20,37]. Additionally, this study excluded PwPD with severe cognitive deficits, which are common in PD and could impact system usability. Individuals with significant cognitive impairment may have different usability needs and may require additional support when using such a system in a home environment, such as a more detailed user manual or video instructions. Moreover, using a tablet interface with a minimalistic user interface (UI) design could simplify the presentation of content and enhance usability for these users.

The long-term usability and reliability of the device in a home setting, as well as the motivation of users to engage with it, remain uncertain and warrant investigation in future longitudinal studies. However, gamification of the exercises or visualizing progress over time within the app could help maintain the patient’s motivation. Furthermore, future studies should assess the device’s potential therapeutic effects on improving fine motor skills. This could be achieved through a prospective, longer-term interventional study, in which quantitative fine motor skill tests, such as the nine-hole peg [38] test or the coin rotation task [39], are conducted and compared pre and post intervention.

## 5. Conclusions

The present study demonstrates high usability and satisfaction with the SSE system, equipped with a newly developed app, both in healthy subjects and in PwPD. The system was well accepted. The quality of information and user interface enabled participants to exercise independently, resulting in a high overall level of satisfaction. The SSE and app showed promise for evaluation in home settings as a part of future long-term prospective training programs. Additionally, the newly developed algorithm effectively detects exercise repetition rates but requires further refinement to enhance accuracy for specific pinch grip exercises.

## Figures and Tables

**Table 1:** Overview of the exercises performed during the study and the corresponding sensors used to measure key movement parameters. FSR = force sensing resistor; SSE = Smart Sensor Egg.

Exercise	Image	Description	Sensor
1	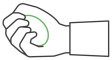	Compression and release of the SSE with one hand	FSR
2	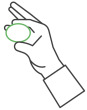	Compression and release of the SSE with the index finger	FSR
3	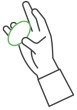	Compression and release of the SSE with the ring finger	FSR
4	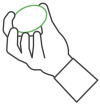	Rotation of the SSE around the yaw axis with one hand	Gyroscope, Accelerometer
5	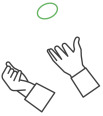	Tossing and catching the SSE from one hand to the other	Accelerometer
6	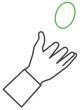	Tossing and catching the SSE with one hand	Accelerometer

**Table 2:** People with Parkinson’s Disease (PwPD) and healthy subjects (HS)—clinical and demographic characteristics.

	PwPD (*n* = 7)	HS (*n* = 7)	*p*-Value
Age, y	66 (64.5–73.5)	64 (51.5–72.5)	0.522
Sex (m/f), n	6/2	6/2	1.000
MoCa ^1^	26 (26–27.5)	-	-
Disease duration, y	4 (3.5–10)	-	-
MDS-UPDRS-III	22 (21–33.5)	-	-
Hoehn and Yahr Stage	2 (2–2.5)	-	-

^1^ Note: MoCA assessment was not conducted for one patient. All values are presented as median and interquartile ranges (Q1–Q3); y = years; m = male; f = female; n = number of participants; MoCA = Montreal Cognitive Assessment; and MDS-UPDRS-III = Movement Disorders Society Unified Parkinsons Disease Rating Scale.

**Table 3:** Difference between repetitions counted by the supervisor and the algorithm, with “-” for no difference. Light red = Exercises not performed correctly, e.g., due to incorrect hardware handling or insufficient strength to reach the required threshold for counting a repetition to be counted. Blue = The exercise was performed correctly, but the repetition recognition algorithm was incorrect.

	Differenz					
**Participant**	**Exercise 1**	**Exercise 2**	**Exercise 3**	**Exercise 4**	**Exercise 5**	**Exercise 6**
PwPD1	-	1	-	5	-	-
PwPD2	-	2	2	-	2	-
PwPD3	5	4	1	4	-	-
PwPD4	3	23	9	-	1	-
PwPD5	-	1	12	-	2	-
PwPD6	10	15	22	-	5	3
PwPD7	-	-	17	3	-	-
HS1	-	-	-	-	-	-
HS2	-	-	7	-	-	-
HS3	2	-	7	-	-	-
HS4	-	-	4	-	-	-
HS5	-	-	-	-	-	-
HS6	-	1	2	6	-	1
HS7	4	-	-	-	-	2

## Data Availability

The datasets utilized and analyzed in this study are available from the corresponding author, T.V., upon reasonable request, while ensuring compliance with ethical and sensitivity considerations.

## Supplementary Materials

The following supporting information can be downloaded at https://www.mdpi.com/article/10.3390/s25041051/s1, PDF S1: Instructions; Video S1: Smart Sensor Egg Demo.

## Appendix A. Repetition Count Overview

To assess the performance of the algorithms, their counted repetitions were compared with those recorded by the supervisor. Table A1 provides a detailed comparison, listing the repetitions counted by the supervisor (TGT), the algorithm (ACT), and the resulting difference (DIFF) for each exercise and participant.

**Table A1:** Overview of the repetitions counted by the supervisor (actually performed) and recognized by the algorithms for each patient and exercises. Light red = exercises not performed correctly, e.g., due to incorrect hardware handling or insufficient strength to reach the required threshold for counting a repetition to be counted. Blue = the exercise was performed correctly, but the repetition recognition algorithm was incorrect. “TGT” for targeted refers to the repetitions counted by the supervisor, while “ACT” refers to the actual repetitions detected by the algorithm. “DIFF” shows the difference between repetitions counted by the supervisor (TGT) and actual repetitions detected (ACT) by the algorithms.

	E1			E2			E3			E4			E5			E6		
**Participant**	**TGT**	**ACT**	**DIFF**	**TGT**	**ACT**	**DIFF**	**TGT**	**ACT**	**DIFF**	**TGT**	**ACT**	**DIFF**	**TGT**	**ACT**	**DIFF**	**TGT**	**ACT**	**DIFF**
PwPD1	17	17	0	19	20	1	21	21	0	6	1	5	25	25	0	23	23	0
PwPD2	16	16	0	21	23	2	22	24	2	6	6	0	23	25	2	22	22	0
PwPD3	20	25	5	19	23	4	23	24	1	4	0	4	27	27	0	23	23	0
PwPD4	18	21	3	23	0	23	19	10	9	7	7	0	25	24	1	23	23	0
PwPD5	17	17	0	28	27	1	26	38	12	7	7	0	24	26	2	24	24	0
PwPD6	10	20	10	15	0	15	20	42	22	3	3	0	16	21	5	19	22	3
PwPD7	0	0	0	0	0	0	22	39	17	5	2	3	31	31	0	23	23	0
HS1	41	41	0	29	29	0	43	43	0	10	10	0	23	23	0	22	22	0
HS2	24	24	0	42	42	0	36	43	7	5	5	0	29	29	0	29	29	0
HS3	20	22	2	23	23	0	16	23	7	12	12	0	23	23	0	21	21	0
HS4	15	15	0	27	27	0	31	27	4	13	13	0	23	23	0	25	25	0
HS5	17	17	0	19	19	0	17	17	0	7	7	0	27	27	0	29	29	0
HS6	17	17	0	21	22	1	16	18	2	6	0	6	25	25	0	18	19	1
HS7	20	24	4	19	19	0	20	20	0	11	11	0	25	25	0	23	25	2

## Appendix B. Example Signal

The algorithm used for exercises 1, 2, and 3 is based on a threshold methodology. A repetition is counted when the signal from the force sensing resistor exceeds a predetermined threshold during the compression phase of the Smart Sensor Egg and then falls below that threshold when the pressure is released.

In some cases, insufficient force may only barely exceed the threshold, combined with reduced sensorimotor control, which can cause fluctuations near the threshold. This may result in multiple crossings of the threshold within a single repetition (Figure A1). However, when the force applied clearly surpasses the threshold, the algorithm can accurately detect just one repetition. If there are no tremors, the algorithm continues to function effectively, even if the applied force only slightly meets the threshold requirement.
