# Supplementary material for: A Usability Pilot Study of a Sensor-Guided Interactive System for Dexterity Training in Parkinson’s Disease
Source: Sensors (Basel). 2025 Feb 10;25(4):1051. doi: 10.3390/s25041051 (PMC11859258; doi:10.3390/s25041051)

Bedienungsanleitung SmartEgg App

Bitte lese zuerst die ganze Anweisung durch und führe sie erst danach aus.

1. Entsperre das iPhone/Android Smartphone und klicke auf die App SmartEgg. Bei Starten der App öffnet sich der Ladebildschirm. Das SmartEgg verbindet sich automatisch mit der App. Ist die Verbindung aufgebaut, leitet das App automatisch weiter. Falls sich das App nach ca. 20s nicht weiterleitet, drücke auf Beenden und Starte die App neu.

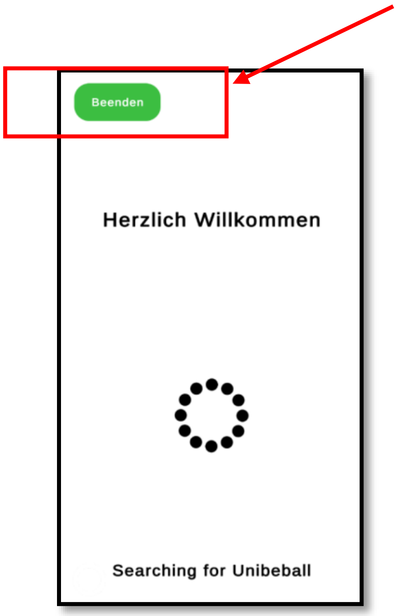

2. Nun wird in den Einstellungen das Trainingsprogramm zusammengestellt und die Übungsdauer definiert. Drücke dafür auf **«Einstellungen».**

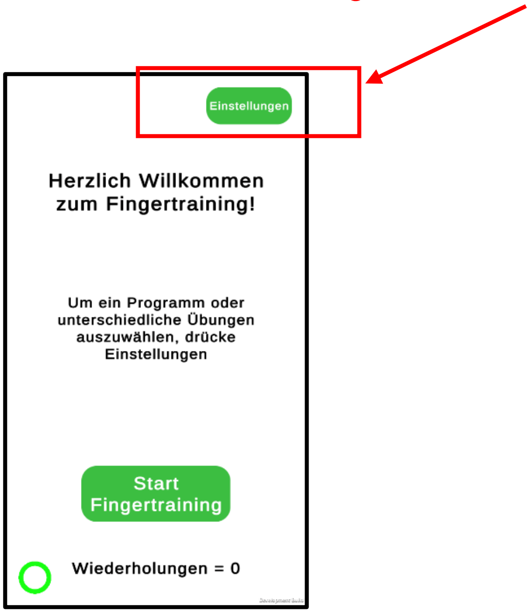

3. Um das Trainingsprogramm zusammen zu stellen, drücke auf **«Übungen auswählen»**.

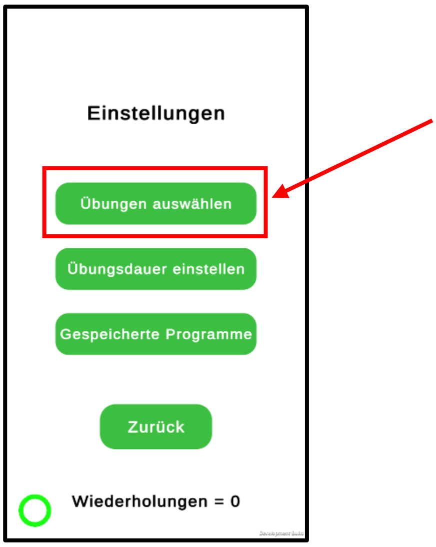

4. Kontrolliere ob **alle** Übungen angewählt sind (Hacken gesetzt), wenn nicht, wähle **alle** Übungen aus. Sind alle Übungen ausgewählt, drücke auf **«Übungen auswählen»**.

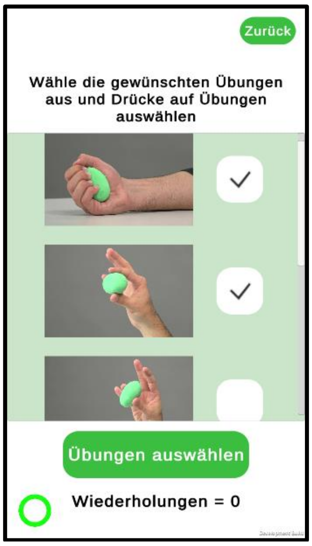

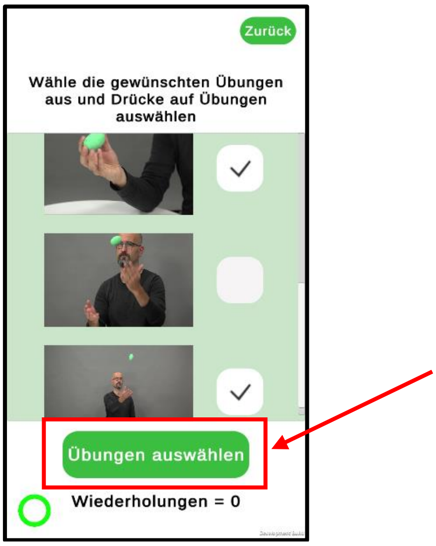

5. Als nächstes wird die Übungsdauer eingestellt. Drücke dafür auf **«Übungsdauer Einstellen»**

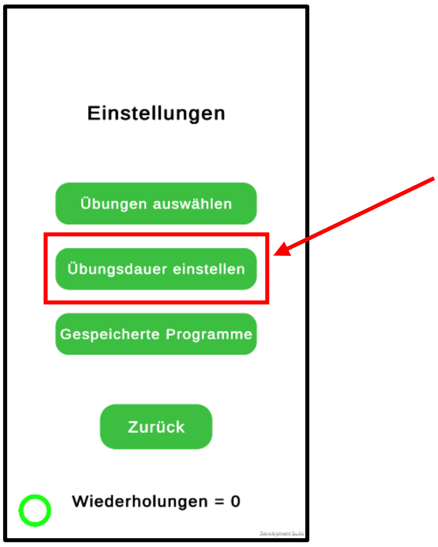

6. Wähle eine Übungsdauer von 30 Sekunden. Drücke dafür auf **«30 Sekunden».**

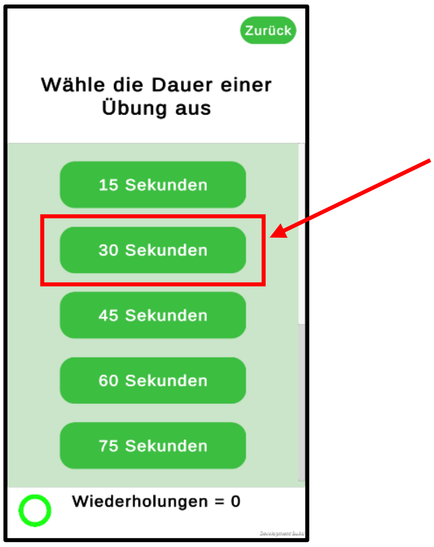

7. Nun sind alle Einstellungen durchgeführt. Drücke auf **«Zurück»**, um wieder zum Start zu gelangen.

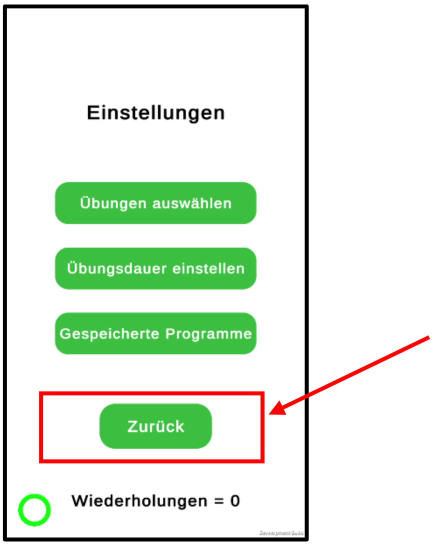

8. Jetzt kann das Fingertraining beginnen. Um das Training zu starten, drücke auf **«Start Fingertraining».**

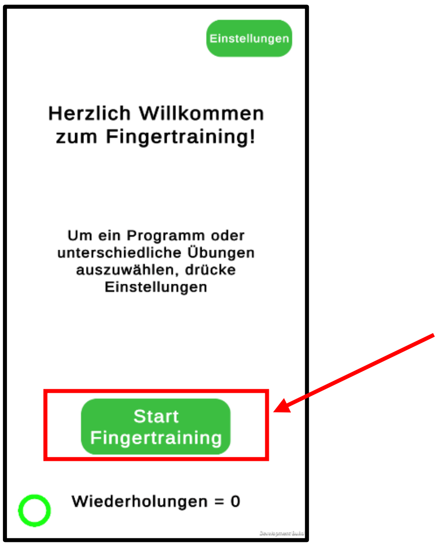

9. Schaue zuerst im Video, wie die Übung durchgeführt wird. Wenn du bereit bist, drücke auf **«Start»** und führe so lange die Übung durch, bis der Countdown fertig ist. Du wirst automatisch weitergeleitet.

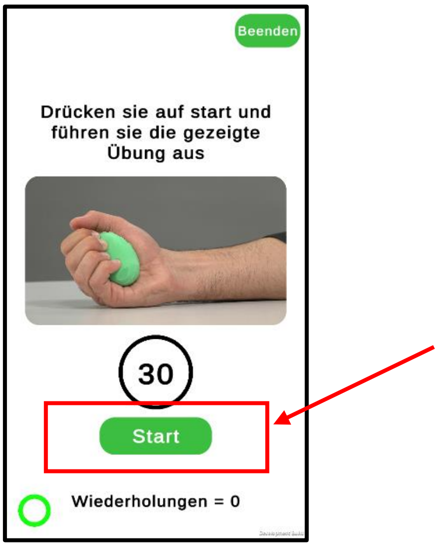

10. Ist der Countdown fertig, erscheint folgender Screen. Dieser Zeigt die Anzahl Wiederholungen, die durchgeführt wurden. Wenn du wieder bereit bist, drücke auf **«Nächste Übung»**. Es erscheint erneut das Bild von Punkt 10, mit einem neuem Übungsvideo. Dies Wiederholt sich, bis alle Übungen durchgeführt sind.

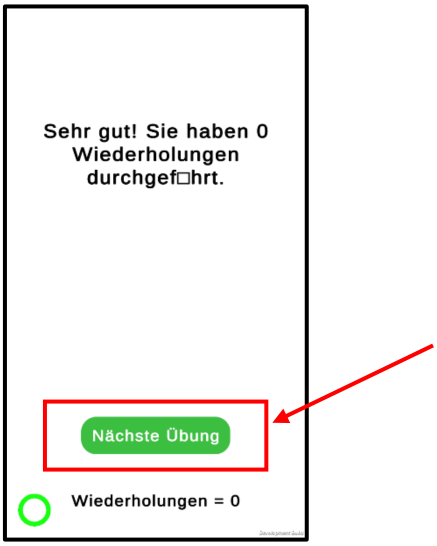

11. Sind alle Übungen durchgeführt, erscheint folgendes Bild. Das Finger Training ist beendet. **Melde die angezeigte Nummer dem Betreuer**.

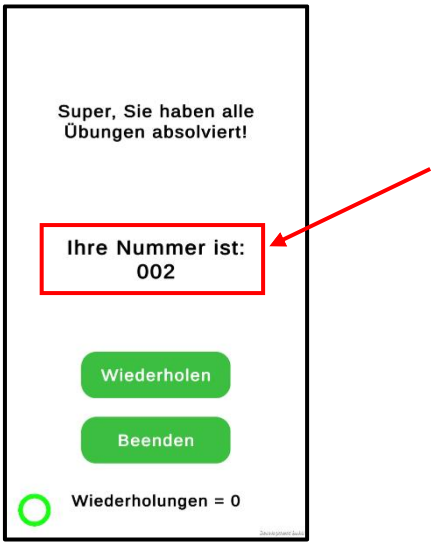

12. Um die App zu beenden, drücke auf **«Beenden»**.

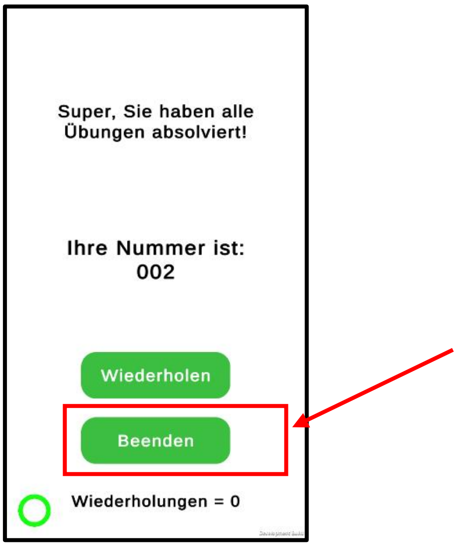

Supplement: Supplementary file 1 [file sensors-25-01051-s001.zip › Supplementary/Bedienungsanleitung_SmartEgg_App.pdf]
